# Human Pluripotent Stem Cells Go Diabetic: A Glimpse on Monogenic Variants

**DOI:** 10.3389/fendo.2021.648284

**Published:** 2021-05-17

**Authors:** Sandra Heller, Michael Karl Melzer, Ninel Azoitei, Cécile Julier, Alexander Kleger

**Affiliations:** ^1^ Department of Internal Medicine I, Ulm University Hospital, Ulm, Germany; ^2^ Department of Urology, Ulm University Hospital, Ulm, Germany; ^3^ Université de Paris, Institut Cochin, INSERM U1016, CNRS UMR-8104, Paris, France

**Keywords:** pluripotent stem cells, diabetes, monogenic variants, Maturity Onset of Diabetes in the Young, type 2 diabetes, type 1 diabetes

## Abstract

Diabetes, as one of the major diseases in industrial countries, affects over 350 million people worldwide. Type 1 (T1D) and type 2 diabetes (T2D) are the most common forms with both types having invariable genetic influence. It is accepted that a subset of all diabetes patients, generally estimated to account for 1–2% of all diabetic cases, is attributed to mutations in single genes. As only a subset of these genes has been identified and fully characterized, there is a dramatic need to understand the pathophysiological impact of genetic determinants on *β*-cell function and pancreatic development but also on cell replacement therapies. Pluripotent stem cells differentiated along the pancreatic lineage provide a valuable research platform to study such genes. This review summarizes current perspectives in applying this platform to study monogenic diabetes variants.

## Introduction

Diabetes, as one of the major diseases in industrial countries, affects over 350 million people worldwide. Type 1 (T1D) and type 2 diabetes (T2D) are the most common forms. T2D accounts for most diabetes cases and is a multifactorial metabolic disease where insulin deficiency is caused by insulin resistance in target organs and pancreatic *β*-cell failure. The current diabetes classifications are insufficient to explain the large clinical and biological variability of diabetes, suggesting an unrecognized level of heterogeneity (1). T1D is described as a chronic autoimmune disease against insulin-producing *β*-cells leading to hyperglycemia. T1D results from the combination of multiple factors, including environment, genes, and a prominent role of the immune system. Genetic studies have long recognized that mutations of the human leukocyte antigens (HLAs) within the Major Histocompatibility Complex (MHC) represent major genetic risk factors in T1D (2, 3). More recently, genome-wide association studies (GWAS) and candidate gene approaches have identified more than 50 other loci contributing to T1D risk, including *INS, PTPN22, CTLA4*, and *GLIS3* genes (4, 5). In addition, mutations in several genes, such as *AIRE, FOXP3, and STAT* (6, 7), may cause rare monogenic forms of autoimmune diabetes.

Similarly, genetic studies of T2D identified many single nucleotide polymorphisms (SNPs) associated with T2D risk, which are located near several functionally relevant genes such as *PPARG* (8), *WFS1* (9), *KCNJ11* (10), *KLF14* (9), *ANK1* (11), *INS* (12), *HNF1A* (9), *HNF1B* (13), and *GLIS3* (14). In addition to genetic predisposition, environmental factors and epigenetic changes are influencing the pathophysiology of T2D, which may contribute to the additional variance in susceptibility.

Overall, genetic studies of T1D and T2D resulted in the identification of many disease-associated variants, most of which, with the exception of the HLA locus for T1D, contribute to a small increase in disease risk (5, 15, 16). These studies have provided valuable information on putative genes and the mechanisms involved in diabetes. For example, many genes identified by T2D GWAS are expressed in human islets (17) and may regulate *β*-cell mass and function (18). While a large proportion of T1D susceptibility genes are surprisingly not related to the immune system (19), studies from D. Eizirik’s group have also shown that >60% of these genes are expressed in *β*-cells, and their expression is affected upon exposure to cytokines, viruses, and double-stranded RNA, a by-product of viral infection, in human and rodent *β*-cells (19–21). Altogether, these studies suggest an essential role of mechanisms acting at the level of *β*-cells in the etiology of both T1D and T2D (19–21).

Interestingly, several of these genes are involved in both monogenic diabetes (rare variants) and multifactorial diabetes (frequent variants). This is the case of the insulin gene (*INS*, monogenic neonatal diabetes, Maturity Onset of Diabetes in the Young (MODY) and multifactorial T1D), as well as *KCNJ11, WFS1, HNF1A*, *HNF1B* (neonatal, syndromic, or MODY monogenic diabetes, and multifactorial T2D) and *GLIS3* (neonatal monogenic diabetes and multifactorial T1D and T2D). This plethora of genes involved in common multifactorial and rare monogenic forms of diabetes suggests that some disease mechanisms and biological pathways may be shared between different forms of diabetes. The identification and detailed study of genes responsible for monogenic diabetes are therefore extremely valuable to investigate important genes and pathways involved in both monogenic diabetes and common forms of diabetes. Noteworthy, it has become evident most recently that a subset of all diabetes patients, generally estimated to account for 1–5% of all diabetic cases, is attributed to mutations in single genes (22, 23).

As only a subset of these genes has been identified and fully characterized, there is a dramatic need to understand the pathophysiological impact of genetic determinants on *β*-cell function and pancreatic development but also on cell replacement therapies. Although islet transplantation can lead to insulin-independency of diabetic patients for 5 years or longer, this therapeutic option is only accessible for a rare number of patients due to the limited number of cadaveric human islets and complex handling (24). On the other hand, the use of human pluripotent stem cells [hPSCs, induced pluripotent (iPSC) and embryonic stem cells (hESC)] may bypass this need by generating mature *β*-cells *in vitro* upon improving the current protocols of *β*-cell generation.

**Figure 1:**
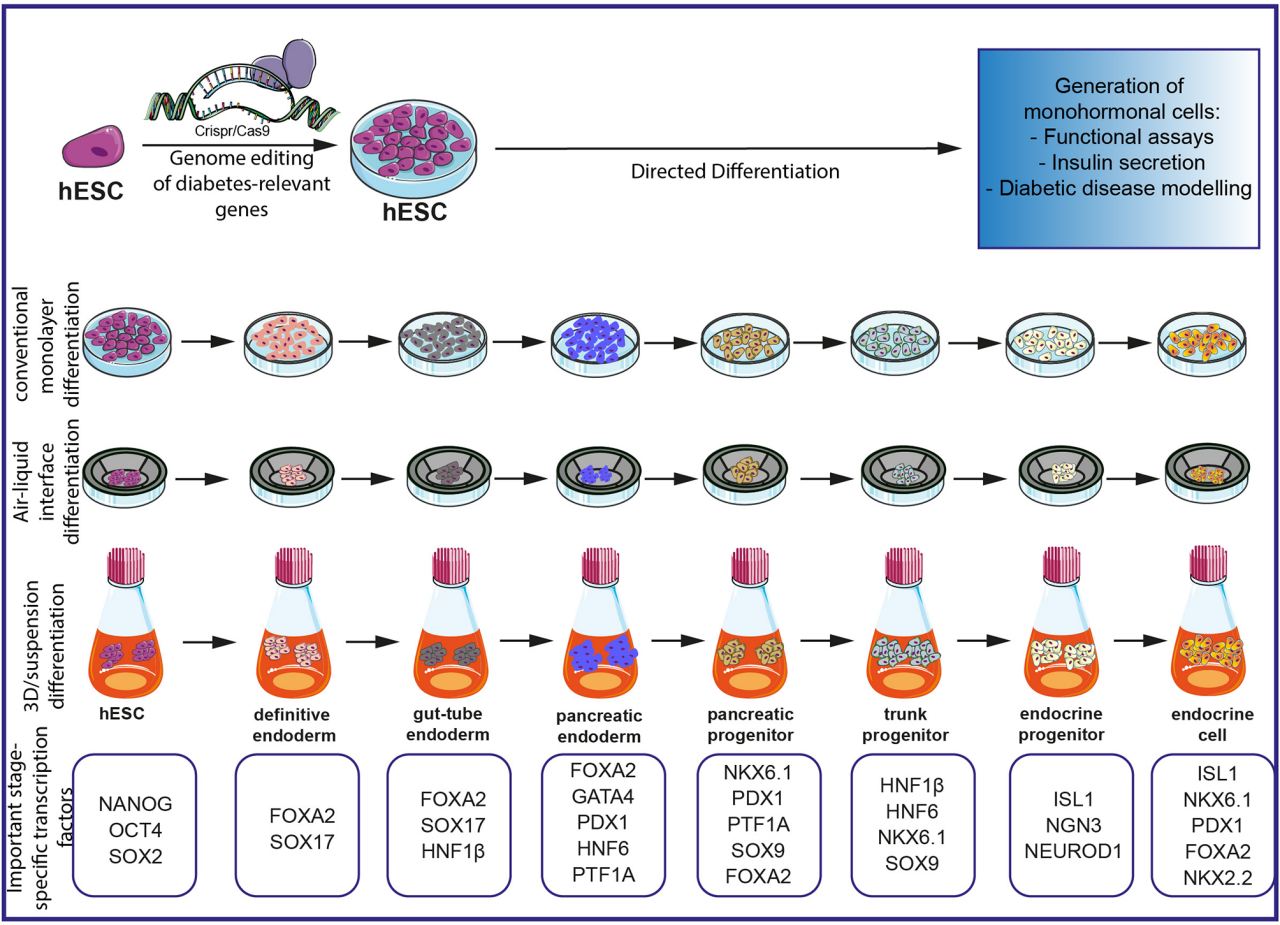
Schematic overview of currently existing protocols for the investigation of diabetes on an hPSC-based platform. Gene editing with CRISPR/Cas9 allows precise editing of diabetes-relevant genes and generation of hESC for further differentiation experiments. Different differentiation protocols allow the generation of monohormonal cells by passing through different milestones during embryonic development. Important stage-specific transcription factors are indicated below the schematics. Subsequent analysis of monohormonal *β*-cells, including insulin secretions assays, can be performed and generate hypotheses about the influence of specific genetic variants. The figure was modified from Smart Servier Medical Art (https://smart.servier.com/) under a Creative Common Attribution 3.0 Generic License.

PSCs have been used as a relevant model system to elucidate pathophysiological mechanisms in diseases such as diabetes, blood disorders, defined neurological disorders, and genetic liver disease (25–27). Induced pluripotent stem cells (iPSCs) allow dissecting monogenic human disease mechanisms (28) as well as mechanisms of genetically complex human disorders such as schizophrenia (29). This opens promising perspectives in both regenerative medicine but also in drug development to screen for innovative, “druggable” targets (30) and to develop *ex vivo* gene-targeting therapies (28). Given the still high intra- and interpatient variability of patient-derived iPSCs, controls are the key for a precise analysis (31). Recent advances in the development of genomic editing tools such as the Zinc-finger or clustered regularly interspaced short palindromic repeats (CRISPR)/Cas9 technology have further revolutionized this research field. Now researchers can precisely modify a human pluripotent stem cell genome with (i) high efficiency, (ii) on a single-base resolution, (iii) without altering the pluripotent capacity, and (iv) with negligible off-target effects to provide isogenic controls and to facilitate data interpretation. In turn, these recent tools represent novel state-of-the-art disease-in-a-dish models and will pioneer research fields aiming to understand also the mechanisms underlying monogenic diseases (32–35). Human pancreatic disease modeling is highly dependent on reliable and efficient differentiation protocols for human PSCs. We and others have recently challenged the currently existing protocols (36), first, to optimize the step toward pancreatic progenitor cells (37), second, to drive maturation in a 3D environment (38–40), and third, to increase yields of true monohormonal *β*-cells (41–44). In turn, optimized differentiation platforms now allow for appropriately modeling complex pancreatic diseases such as diabetes (45). A schematic overview of currently available disease modeling tools for diabetes employing hPSCs is presented in **Figure 1**.

## Maturity-Onset Diabetes of the Young

So far, 14 subtypes of maturity-onset diabetes of the young (MODY) have been described to be caused by mainly heterozygous dominant mutations in genes for pancreas-specific transcription factors as well as enzymes, hormones, and ion channels (46, 47). These mutations impair endocrine function at various levels ranging from alterations in development, glucose sensing, synthesis, and storage of insulin to inappropriate secretion of insulin in *β*-cells. The most frequently identified mutations are located in the *HNF4A* gene (MODY1) (48) with a frequency of 4–10% (49–51), in the *GCK* gene (MODY2) (52) with 30–60% (49–51, 53) and in the *HNF1A* gene (MODY3) (54) with 30–50% (49–51) depending on the study population. MODY1 patients are particularly characterized by defective glucose-stimulated insulin secretion possibly caused by disrupted gene expression playing a role in glucose transport and glycolysis (48, 55, 56). MODY2 due to glucokinase deficiency often results in mild hyperglycemia during early life (57). These patients have a defect in glucose-stimulated insulin secretion caused by impaired glucose sensitivity in *β*-cells. MODY3 patients develop *β*-cell dysfunction and hyperglycemia caused by impairment of glucose-dependent insulin secretion (58).

In addition, rare MODY cases [accounting for up to 6% of all MODY forms (49)] have been diagnosed with mutations in *PDX1* (59)*, HNF1B* (60)*, NEUROD1* (61, 62)*, KLF11* (63)*, CEL* (64)*, PAX4* (65)*, INS* (66)*, BLK* (67)*, ABCC8* (68)*, KCNJ11* (69), and *APPL1* (70) (known as MODY4-14).

mpaired functions of proteins caused by pathogenic variants can vary depending on the nature of the mutation, therefore causing a spectrum of clinical manifestations. Patients harboring heterozygous *HNF1B* mutations suffer from MODY, but may also feature pancreas exocrine dysfunction as well as kidney and liver abnormalities (71) and vaginal and uterine malformation (72). Few specified cases of *NEUROD1* mutations are characterized mainly by early onset diabetes (61, 62, 73), but patients with neurological defects such as pituitary gland hypoplasia, growth hormone deficiency, epilepsy, and intellectual disability have also been described (74, 75). *CEL* mutations cause early onset diabetes associated with exocrine pancreatic dysfunction and chronic pancreatitis (76, 77). In addition to the diabetic phenotype in patients with *PAX4* mutations, diabetic complications such as retinopathy and nephropathy have been observed (65, 78). MODY-causing *INS* mutations have been associated with early onset diabetes as well as ketoacidosis in some cases (79), whereas rare cases with *BLK* mutations have also been associated with overweight (67).

No other clear clinical manifestation besides diabetes has been described for patients with heterozygous mutations in *KLF11* (MODY7), *ABCC8 (*MODY12), *KCNJ11 (MODY13*), and APPL1 (MODY14). A summary of confirmed MODY-causing mutations as well as of other prominent clinical features is presented in **Table 1**.

**Table 1:** Different MODY forms, including their frequencies, affected genes, and potential other prominent clinical manifestations are presented.

MODY form	Affected gene	Frequency	Potential prominent additional clinical manifestations besides diabetes and its complications	Affected gene investigated using hESC	Affected gene investigated using hiPSC
MODY1	*HNF4A*	4–10%	Not relevant	No	Yes (80–82)
MODY2	*GCK*	30–60%	Not relevant	No	Yes (80, 83)
MODY3	*HNF1A*	30–50%	Not relevant	Yes (84, 85)	Yes (80)
MODY4	*PDX1*	Rare	Pancreatic agenesis and miscarriages	Yes (86)	Yes (87–89)
MODY5	*HNF1B*	Rare	Exocrine pancreatic dysfunction, kidney and liver abnormalities, vaginal aplasia, and uterus hypoplasia	No	Yes (80, 90, 91)
MODY6	*NEUROD1*	Rare	Neurological defects including pituitary hypoplasia, growth hormone deficiency, epilepsy, and intellectual disability	No	No
MODY7	*KLF11*	Rare	Nothing else described	No	No
MODY8	*CEL*	Rare	Exocrine pancreatic dysfunction, chronic pancreatitis	No	Yes (80)
MODY9	*PAX4*	Rare	Not relevant	Yes (92)	No
MODY10	*INS*	Rare	Not relevant	No	Yes (93)
MODY11	*BLK*	Rare	Overweight	No	No
MODY12	*ABCC8*	Rare	Nothing else described	Yes (94, 95)	No
MODY13	*KCNJ11*	Rare	Nothing else described	Yes (96)	Yes (97)
MODY14	*APPL1*	Rare	Nothing else described	No	No

Furthermore, a statement about the current research of the respective mutations or MODY forms, including hESC and hiPSC, is included.

Noteworthy, the homozygous status for mutations in several MODY genes has been found to lead to extreme clinical presentations, contrasting with the less severe early onset diabetes observed in heterozygous carriers. For example, homozygous mutations in the *PDX1* gene result in early onset diabetes associated with pancreatic agenesis and maternal miscarriages (59, 98).

A subset of patients with MODY-like phenotype doesn’t carry any mutation in the known MODY genes, suggesting the involvement of additional genes. The identification of these additional genes responsible for rare MODY forms is now facilitated by the availability of large databases of diabetic cases and control cohorts that enable increased efficiency to detect novel genes with rare contributing variants (including MODY-like effects) (99), compared to earlier studies with smaller sample size (100). In addition, the availability of large databases of control subjects (*e.g.*, gnomAD, TOPMED) provides now the possibility to estimate the frequency of rare coding variants in candidate genes, hence allowing for efficient association and burden-testing for rare monogenic contributions, such as MODY. Consequently, recent studies identified *RFX6* as a novel MODY gene (101) and *WFS1, PPARG*, and *GLIS3* have recently been proposed as potential candidates for these rare MODY forms (101–103).

Taken into consideration the overlap in genes involved in common multifactorial and rare monogenic forms of diabetes, the specific analysis of monogenic pathogenic variants can therefore reveal novel interaction partners and gene targets that might be helpful to better understand the mechanisms involved in the onset of T1D and T2D.

## Permanent Neonatal Diabetes Mellitus

Permanent neonatal diabetes mellitus (PNDM) is the second form of monogenic diabetes. It is characterized by hyperglycemia and partial or complete insulin deficiency in patients in the first 6 months postnatal (104, 105). Moreover, patients with PNDM may suffer from intrauterine growth retardation, glycosuria, ketoacidosis, failure to thrive as well as various clinical features depending on the gene. Mutations in more than 20 genes with monogenic contribution important for *β*-cell development have been identified to cause PNDM (46). Treatment of PNDM includes oral sulfonylureas or insulin therapy and may require pancreatic enzyme replacement for infants with pancreatic aplasia or hypoplasia.

Some genes, including *ABCC8* (106), *GCK* (107)*, INS* (108)*, KCNJ11* (109), and *PDX1* (110) may alternatively cause PNMD or MODY, with various severity and clinical features depending on the gene, nature of the mutation, and genotype (homozygous or heterozygous). Common variants in these genes may also be associated with multifactorial T1D or T2D.

In addition to diabetes, *KCNJ11-*PNMD patients may also have neurological features such as developmental delay and epilepsy (DEND syndrome) (111). Similarly, pathogenic homozygous *PDX1*-PNDM patients have pancreatic agenesis and pancreatic hypoplasia leading to exocrine pancreatic insufficiency (110, 112). Pancreatic agenesis is furthermore caused by homozygous mutations in another pancreatic transcription factor, *PTF1A* (113). Here, PNDM patients additionally suffer from severe intrauterine growth retardation, cerebellar agenesis, and neurological dysfunction.

In addition, PNDM may manifest in the context of specific syndromes. Homozygous mutations in *EIF2AK3* cause Wolcott–Rallison syndrome, characterized by PNDM, exocrine pancreas dysfunction, and abnormalities such as liver failure, developmental delay, and epiphyseal dysplasia (114). Inactivating *GATA4* variants can induce pancreatic agenesis or hypoplasia, causing PNDM but also lead to extrapancreatic symptoms such as cardiac and neurodevelopmental abnormalities (115). Similarly, *GATA6* mutations cause pancreatic agenesis leading to PNDM, together with abnormalities of the heart, biliary tract, and gut development (116). Homozygous mutations in *GLIS3* cause PNDM together with congenital hypothyroidism associated with congenital glaucoma, hepatic fibrosis, and polycystic kidneys (117). In addition to neonatal diabetes, *NEUROD1* mutations cause cerebellar hypoplasia, sensorineural deafness, and visual impairment (118), whereas *NEUROG3* mutations affect intestinal development leading to congenital malabsorptive diarrhea (119–121). Mutations in *PAX6*, encoding a transcription factor involved in *β*-cell development as well as eye and brain development, cause neonatal diabetes combined with abnormalities of the central nervous system and visual system (*e.g.* microencephaly, optic nerve defects, microphthalmia) (122). In addition to PNDM, mutations in the transcription factor *RFX6* cause pancreatic hypoplasia, intestinal atresia, and gall bladder hypoplasia (123, 124). Patients with Wolfram syndrome caused by mutations in *WFS1* suffer from early onset diabetes as well as optic atrophy, deafness, ataxia, and dementia (125). Other neonatal diabetes syndromes have been described for mutations in *SLC19A2* (associated with thiamin-responsive megaloblastic anemia, neurological disorders, cardiac abnormalities, and deafness) (126, 127), *MNX1* (associated with growth retardation, delayed central nervous system development, hypoplastic lungs, renal maldevelopment, skeletal dysplasia) (128), *NKX2.2* (further leading to growth retardation, delayed central nervous system development, constipation) (128), and *IER3IP1* (additional microcephaly, CNS maldevelopment) (129). Furthermore, some mutations in the glucose transporter *SLC2A2* can cause neonatal diabetes prior to the Fanconi–Bickel syndrome associated with glycosuria, galactosemia, aminoaciduria, proteinuria, hepatomegaly, as well as glucose and galactose intolerance (130, 131). Franco et al. recently showed that mutations in *YIPF5* cause neonatal diabetes associated with microcephaly and epilepsy (132).

Interestingly, an overlapping phenotype between PNDM and autoimmune T1D was observed for a patient with an activating mutation in the *STAT3* gene (133). Although the autoimmune-mediated destruction of *β*-cells was prominent Saarimäki-Vire et al. revealed an additional mechanism (PNDM) due to the observed pancreatic hypoplasia (134).

An overview of all described genes leading to the development of PNDM if affected by mutations is presented in **Table 2**.

**Table 2:** Overview of mutations in genes that can lead to PNDM.

Affected gene in PNDM	Affected gene also described for MODY	Part of a syndromic phenotype	Potential prominent additional clinical manifestations	Affected gene investigated in hESC	Affected gene investigated in hiPSC
*ABCC8*	MODY12	No	Nothing else described	Yes (94, 95)	No
*EIF2AK3*	No	Yes, Wolcott-Rallison syndrome	Exocrine dysfunction, acute liver failure, developmental delay, epiphyseal dysplasia	No	No
*GATA4*	No	Yes	Pancreatic agenesis, cardiac, and neurodevelopmental abnormalities	No	No
*GATA6*	No	Yes	Pancreatic agenesis, abnormalities of heart, biliary tract, and gut development	Yes (135, 136)	Yes (135, 136)
*GCK*	MODY2	No	Not relevant	No	Yes (80, 83)
*GLIS3*	Potentially	Yes	Congenital hypothyroidism, congenital glaucoma, hepatic fibrosis, polycystic kidneys, pancreatic exocrine insufficiency, kidney, liver, and biliary dysfunction	Yes (86)	No
*IER3IP1*	No	Yes	Microcephaly, CNS maldevelopment	No	No
*INS*	MODY10	No	Not relevant	No	Yes (93, 137, 138)
*KCNJ11*	MODY13	No	Nothing else described	Yes (96)	Yes (97)
*MNX1*	No	Yes	Growth retardation, delayed central nervous system development, hypoplastic lungs, renal maldevelopment, skeletal dysplasia	Yes (86)	No
*NEUROD1*	MODY6	Yes	Cerebellar hypoplasia, sensorineural deafness, visual impairment	No	No
*NEUROG3/NGN3*	No	Yes	Intestinal maldevelopment with malabsorptive diarrhea	Yes (86, 139)	No
*NKX2.2*	No	Yes	growth retardation, delayed central nervous system development, constipation	No	No
*PAX6*	No	Yes	Abnormalities of the central nervous system and visual system including microencephaly, optic nerve defects, microphthalmia	No	No
*PDX1*	MODY4	Yes	Pancreatic agenesis and miscarriages	Yes (86)	Yes (87–89)
*PTF1A*	No	Yes	Intrauterine growth retardation, pancreatic agenesis, cerebellar agenesis, and neurological dysfunction	Yes (86)	No
*RFX6*	Potentially	Yes, Mitchell–Riley syndrome	Pancreatic hypoplasia, intestinal atresia, and gall bladder hypoplasia	Yes (86)	Yes (140)
*SLC19A2*	No	Yes, Thiamine-responsive megaloblastic anemia	Megaloblastic anemia, hearing loss, neurological disorders, cardiac abnormalities	No	No
*SLC2A2*	No	Yes, Fanconi–Bickel syndrome	Glycosuria, galactosemia, aminoaciduria, proteinuria, hepatomegaly, glucose intolerance, galactose intolerance (130, 131)	No	Yes (141)
*STAT3*	No	Potentially correlated to autoimmune diabetes	Strong autoimmune component of diabetes, pancreatic hypoplasia	No	Yes (134)
*YIPF5*	No	Yes	Microcephaly, epilepsy	Yes (132)	Yes (132)
*WFS1*	Potentially	Yes, Wolfram syndrome	Optic atrophy, deafness, ataxia, and dementia	No	Yes (142, 143)

Additional information of mutations leading to syndromic phenotypes and their characteristics is included. Further statements regarding the relationship to MODY forms and feasibility of hPSC to model the disease development and progression in terms of PNDM are included.

## Modeling Pancreatic Endocrine Development

The detailed pathomechanisms of monogenic diabetes are not yet fully understood since mouse models do not completely recapitulate the human disease phenotype (121, 144, 145), and patient samples such as *β*-cells have very limited availability. Moreover, animal models with a specific knockout of MODY genes show species-specific differences that do not entirely recapitulate the patient phenotype (146–150). Therefore, even more suitable disease models are crucial to develop an adequate therapy.

In the recent years, human pluripotent stem cells have been deployed as a suitable human model system. On the one hand, human embryonic stem cells (hESCs) can be subjected to a directed differentiation protocol to investigate different mechanisms during differentiation of mature pancreatic *β*-cells. Additionally, patient-specific induced pluripotent stem cells (iPSCs) can be generated from materials such as fibroblasts and keratinocytes, allowing to address various genetic backgrounds of patients. Of note, gene-mutated iPSCs show high heterogeneity in terms of differentiation efficiency and are best controlled with isogenic, repaired lines. Furthermore, patient-specific iPSCs are a useful tool for biobanking because of their unlimited expansion capacity. Subsequently, these cells are differentiated *in vitro* into disease-relevant cell types such as pancreatic endocrine cells or β-cells.

To better understand the importance of certain genes in the maturation of *β*-cells, genetic engineering may be performed with hESCs and iPSCs. Here, state-of-the-art gene-editing tools such as zinc-finger nucleases (ZFNs), transcription activator-like effector nucleases (TALENs), and the more recent clustered regularly interspaced short palindromic repeats (CRISPRs)/Cas allow the generation of specific point mutations or gene knockouts (KOs). A potential option of genetic engineering might involve gene correction in iPSCs. This allows the generation of autologous *β*-cells for transplantation that may circumvent immune reaction and donor scarcity.

The generation of pancreatic endocrine cells is achieved by different differentiation protocols. Established protocols try to mimic signaling pathways of *in vivo* embryonic developmental stages by involving different/various combinations of growth factors, cytokines, and small molecules known/reported to guide the stem cells through stages of definitive endoderm, gut-tube endoderm, pancreatic endoderm, pancreatic progenitors, and endocrine progenitors to finally yield mature mono-hormonal endocrine cells (**Figure 1**).

Overall differentiation models of hESCs and hiPSCs provide a versatile tool to study the influence of genetic disorders on *β*-cell development in the human pancreas as well as the embryonic development of the human pancreas itself. Different published protocols whose concepts are described below allow the investigation of multiple facets of *β*-cell maturation during different steps of embryonic development. However, the procedure itself may have a huge influence on the phenotype of differentiated cells. This might lead to the bias that a “good” differentiation protocol can overcome the inherent genotypic features, which would rather underestimate the phenotypic features of a certain genotype. This is, for example, the case for a *GATA6-*mutant iPSC cell line with a severe phenotypic loss of endoderm and *β*-cell differentiation capacity in a differentiation condition involving low levels of retinoic acid. On the other hand, high levels of retinoic acid mask this phenotype (136). The same principle might apply to a “poor” differentiation protocol that may overestimate the phenotypic properties of a certain genotype.

## Generation of Mature Pancreatic *β*-Cells Requires Complex and Sophisticated Differentiation Protocols

During the last decade, differentiation protocols have been adapted to achieve a more mature state of *in vitro* differentiated *β*-cells. Since earlier pancreatic endocrine differentiation protocols in monolayers yield mainly an immature or heterogeneous population of polyhormonal cells lacking robust insulin secretion in response to glucose stimulation, a prerequisite of *β*-cells (36, 151), novel *in vitro* approaches including different culture conditions have been established. Recent protocols include a transition to 3D culture using a suspension culture system with spinner flasks and orbital shaker generating endocrine spheres (41, 42). Alternatively, a switch from the initial culture in monolayer to an air–liquid interphase culture stage promoting basal and apical cell polarity generates even more functionally mature *β*-cells (152) (**Figure 1**).

Air–liquid interphase culture systems require spotting the cells from the pancreatic endoderm stage on filters. Upon formation of small cell clusters, the differentiation is further improved as measured by *NGN3* and insulin expression (43). This transition might help to mimic the natural 3D environment and cell orientation within the developing tissue, thus promoting *in vitro* differentiation (153).

Further progress in *β*-cell maturation was also achieved by reaggregating immature cells into enriched *β*-cell clusters using an insulin-driven fluorescence reporter (154). Veres et al. combined cellular reaggregation and *β*-cell purification using CD49a to enrich endocrine cells and promote functional maturation of *β*-cells able to maintain their identity for several weeks in culture (155).

An alternative approach is to enrich precursor cells in the differentiation process. A recent study demonstrated that enrichment of anterior definitive endoderm with CD177 results in a more homogenous pancreatic progenitor population and subsequent better functional maturation (156).

Moreover, besides optimizing technical conditions for differentiation, the modulation of signaling pathways and cytoskeleton is a promising mean to increase the *β*-cell yield. Inhibition of certain pathways such as ROCKII and WNT using specific inhibitors promoted maturation (157, 158). Hogrebe et al. investigated the role of the actin cytoskeleton in promoting the expression of pancreas-specific transcription factors such as NEUROG3 during differentiation (159). The manipulation of actin polymerization during early developmental stages influences the expression of transcription factors important for the specification of lineage fate in pancreatic progenitors. Depolymerization of the cytoskeleton during endocrine induction further improved the functionality of derived *β*-cells, also allowing for a planar differentiation protocol.

Another strategy to improve differentiation efficacy is to modulate the basic content of cell culture media. Two studies explored metabolic changes during *β*-cell maturation (160, 161). Adaptation of nutrient-sensing *via* mTORC1 signaling during the transition from fetal to adult pancreatic islets can be recapitulated by reduced amino acid content in differentiation media, further advancing cellular insulin content and glucose-stimulated insulin secretion (160). Similarly, epigenomic characterization of primary and *in vitro* differentiated pancreatic cells revealed that entrainment to cycles of fasting and feeding leads to circadian control of genes important for energy and insulin metabolism, further improving *β*-cell function (161).

Protocol improvements resulted in more mature *β*-cells and faster reversal of diabetes after transplantation in mice. However, manifold successful approaches show that regulation of human pancreas development is still not fully understood, and various adaptations to endocrine differentiation protocols are difficult to compare because they use different cell lines and culture methods as well as slightly different functional assays. Therefore, more research is necessary to determine the appropriate combination of culture methods, cytokine and small molecule cocktails, purification markers, and metabolic modifications, generating a protocol that robustly produces the desired pancreatic cell types.

For potential clinical use, several questions/issues regarding the composition of transplanted cells containing only *β*-cells or more than one endocrine cell type, the best transplantation site, and whether transplanted cells benefit from co-transplantation with other cell types such as mesenchymal stem cells need to be answered. Moreover, long-term survival and functionality of transplanted cells exceeding the life span of mice have to be addressed. Additionally, the possibility of teratoma formation from the remaining progenitor cells even after prolonged time has to be eliminated. Encapsulation of cells in suitable biomaterials such as alginates or synthetic polymer hydrogels might not only reduce the risk of tumor formation but also provide protection from the immune rejection of the host, removing the need for lifelong immunosuppression.

Although the latest research significantly improved our knowledge about transcriptional regulation, signaling pathways as well as metabolic adaptation during *in vitro* differentiation and maturation of *β*-cells and paved the way for future clinical use, more research is necessary until *in vitro*-generated pancreatic endocrine cells can be used as potential diabetes therapy.

## Pluripotent Stem Cell Models to Understand Monogenic Diabetes

Although iPSC could be successfully generated from T1D and T2D patients, complex autoimmune reactions, environmental influence, as well as multifactorial genetic factors hampered the intimate recapitulation of pathogenesis (162–164). Despite the complexity of T1D and T2D pathogenesis, some recent approaches have been performed to model T1D using pluripotent stem cells. Co-culture studies of iPSCs derived from T1D patients together with immune cells are one such way to model the mechanisms of T1D *in vitro* (165). Yet, it has to be kept in mind that this kind of model requires additional prerequisites such as environmental factors and complex composition of different immune cell types as recently reviewed by Joshi et al. (166). Modeling T2D *in vitro* is far more complex as many more different pathogenic mechanisms can cause or even interact to promote T2D development, including multiple genetic and environmental factors. This fact makes it even harder to investigate T2D by pluripotent stem cell-based approaches solely *in vitro*.

**Figure 2:**
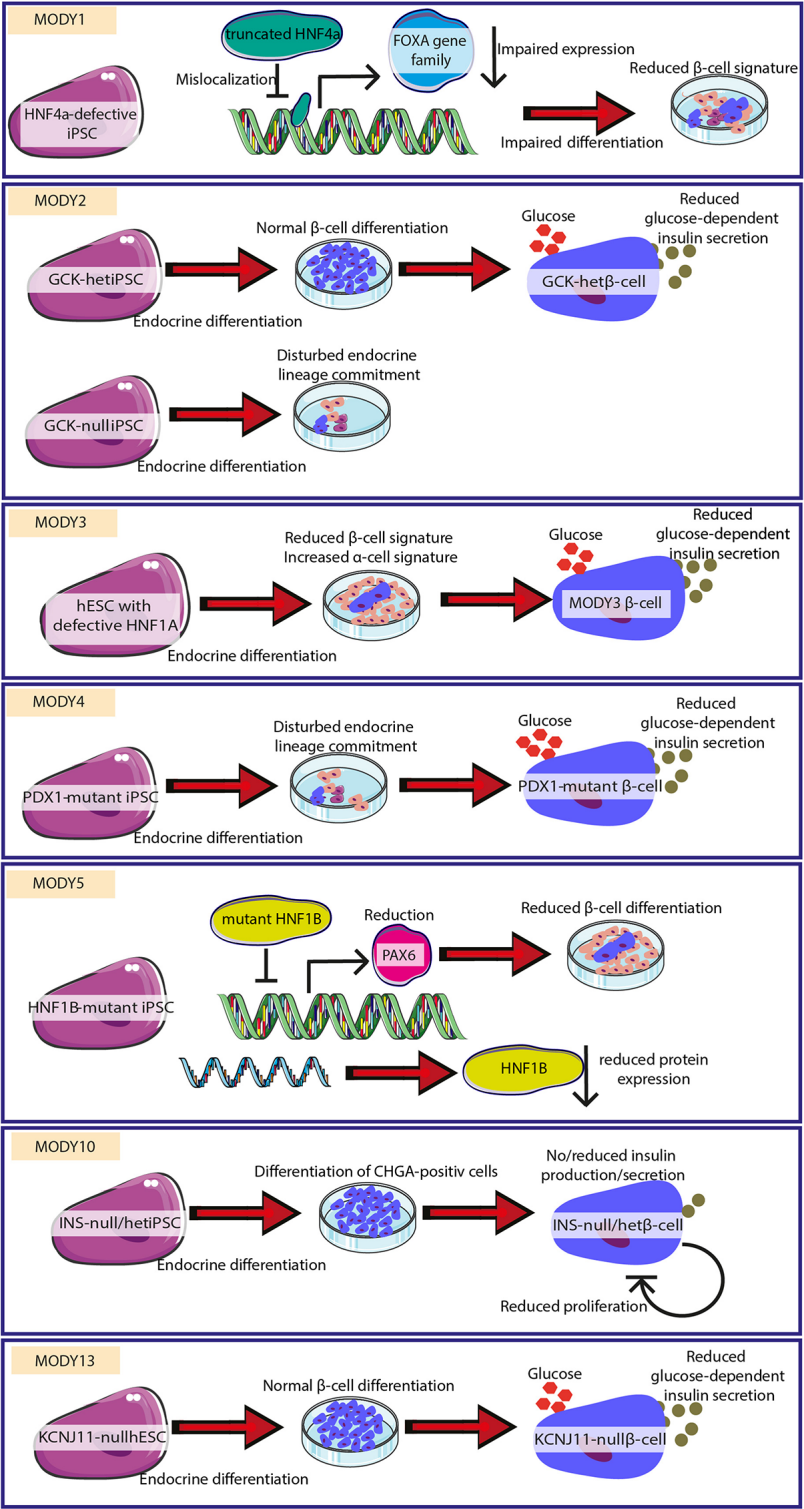
Successful uncovering of pathomechanisms for different MODY forms. Different MODY forms were modeled by employing hESC/hiPSC with respective mutations. Mechanisms leading to monogenic diabetes could be delineated/characterized. In MODY1, mutated HNF4a leads to reduced FOXA gene family expression and impaired *β*-cell signature. MODY2 is characterized by reduced differentiation or reduced glucose-dependent insulin secretion. MODY3 is caused by reduced *β*-cell differentiation and insulin secretion. MODY4 shows reduced endocrine lineage entrance and impaired insulin secretion. MODY5 is caused by diminished *β*-cell differentiation. MODY10 is highlighted by lacking production and secretion of insulin. MODY13 is characterized by impaired glucose-dependent insulin secretion. The figure was modified from Smart Servier Medical Art (https://smart.servier.com/) under a Creative Common Attribution 3.0 Generic License.

Whereas modeling T1D and T2D using PSCs remains challenging due to their complex nature, monogenic alterations leading to a MODY or PNMD diabetes phenotype are ideal to be investigated by PSC-based approaches. The role of specific variants of the respective genes has already been investigated using pancreatic differentiation of pluripotent stem cells. Compared to various genetic and environmental aspects contributing to other diabetes types, single mutations present in monogenic diabetes allow tighter control of the observed phenotypes. Deciphered mechanisms for the development of MODY, which were uncovered using hPSC-based systems, are presented in **Figure 2** (MODY1, 2, 3, 4, 5, 10, 13).

Teo et al. showed that karyotypically normal iPSC expressing pluripotency markers and able to differentiate in all three germ layers could be derived from different MODY patients (MODY1, MODY2, MODY3, MODY5, and MODY8) (80) and can serve as a tool to study the role of the respective genes in pancreatic development. A more detailed study of these MODY1 iPSCs with premature HNF4A protein truncation revealed impaired foregut and hepatopancreatic progenitor development. These events were associated with HNF4A mislocalization and reduced expression of target genes such as the *FOXA* gene family, *HNF1B, PDX1, GATA4*, and *RFX6* (81). In turn, impaired activation of target genes disturbs *β*-cell gene signatures. Prior to that study, iPSCs from MODY1 patients with a nonsense mutation were characterized (82). Here, the patient phenotype was caused by a reduction in levels of functional HNF4A accompanied by increased expression of pancreatic transcription factors and pancreatic hormones as a compensatory mechanism (82).

Understanding the role of transcription factor HNF1A in MODY3, human ESC lacking one or both alleles have been differentiated to study endocrine development (84). HNF1A deficiency increased expression of markers for *α*-cells but reduced expression of *β*-cell markers suggesting a role in endocrine hormone expression. In addition, HNF1A is required for insulin secretion, in line with hyperglycemia observed in patients. Moreover, mutated cells show metabolic defects in glycolysis and mitochondrial respiration, also typical for T2D. This observation, together with the finding that frequent *HNF1A* variants are associated with T2D, suggests a link between mechanisms identified in MODY and in common T2D. Furthermore, iPSCs from a patient harboring heterozygous *HNF1A* mutation generated by non-integrative viral transduction, show a normal karyotype, and express pluripotency factors (85). These cells can be used for further functional analysis of this specific mutation.

Relevant defects in the *GATA6* gene disrupt the endoderm differentiation by decreased cell survival. Later, the pancreas specification and *β*-cell function were identified using iPSC and genome-edited ESC (135, 136). By circumventing the developmental block at the endoderm stage, cell lines with *GATA6* mutations were differentiated with low dose retinoic acid to mimic severe patient phenotype. These cells failed to show normal insulin secretion after glucose stimulation and harbor defective insulin processing (136).

Fibroblasts from patients with heterozygous point mutations in the *PDX1* transactivation domain were successfully reprogrammed to iPSCs and can be used to study diabetes-associated pathomechanisms (87–89). Further pancreatic differentiation reveals that mutations in the *PDX1* transactivation domain disturb the pancreatic endocrine lineage development and result in impairment of the glucose-responsive function of *β*-cells (88).

The analysis of patient-derived iPSCs with *HNF1B* mutations (MODY5) suffering from early onset diabetes and pancreatic hypoplasia revealed a compensatory increase in markers of definitive endoderm and pancreatic transcription factor expression such as PDX1 (90). Additionally, downregulation of transcription factor PAX6, important for islet development (167), may result in the observed patient phenotype (90). Furthermore, iPSCs were generated from a Japanese MODY5 patient with a truncated *HNF1B* variant in order to account for differences in insulin sensitivity and insulin response depending on the genetic background (91). Yabe et al. compared iPSCs derived from healthy and patient skin fibroblasts and detected degradation of mutant mRNA by the nonsense-mediated decay pathway in differentiated patient-derived iPSCs (91).

A more systematic analysis of pancreatic transcription factors *PDX1*, *RFX6*, *PTF1A*, *GLIS3*, *MNX1*, *NGN3*, *HES1*, and *ARX*, partly identified in monogenic variants of MODY and PNDM, characterized the transcriptional control and corresponding defects at several developmental stages (86). This study highlights especially the role of RFX6 in controlling pancreatic progenitor numbers and differences of NEUROG3 requirement in humans and mice. Mutations in *WFS1*, causing Wolfram syndrome, lead to chronic endoplasmic reticulum stress activating the unfolded protein response, which impairs survival of *β*-cells and neurons (168–170). This could be recapitulated using iPSC with *WFS1* variants (142). A recent publication from Maxwell et al. also characterized iPSC from a Wolfram syndrome patient (143). Patient-specific iPSCs harboring a pathogenic variant of *WFS1* were corrected using CRISPR/Cas9 technology. This study used a differentiation protocol with cytoskeletal modification, which significantly improved differentiation efficiency compared to previously tested suspension culture in these cell lines (159). Corrected cells showed higher WFS1 expression and robust insulin secretion, probably benefiting from reduced ER stress and improved mitochondrial respiratory capacity in endocrine cells (143). In turn, further maturation of *in vitro* generated *β*-cells allows better identification of effects also in later stages, additionally providing potential use for *β*-cell replacement therapy.

Homozygous mutations in the insulin gene (*INS*) are known to lead to PNDM. Pancreatic differentiation of patient iPSC results in CHGA-positive endocrine cells expressing *β*-cell markers NKX6.1, PDX1, and MAFA but lacking insulin expression (93). Gene correction rescued the phenotype and prevented diabetes in a streptozotocin mouse model, providing a future tool for patient cell therapy. Another study involved iPSCs generated from patients with neonatal diabetes and heterozygous insulin mutations disturbing proper proinsulin folding (137). Patient-derived iPSCs show normal pancreatic differentiation comparable to corrected isogenic iPSC but have reduced insulin expression. Moreover, *INS* mutation increases ER stress and hampers proliferation of *β*-cells but without increased apoptosis promoting diabetes development in patients. In addition, fibroblasts from a PNDM patient harboring an intronic *INS* mutation have been efficiently generated and may serve as a diabetes model to characterize the expected aberrant splicing (138).

Another study characterized iPSCs from MODY2 patients with a heterozygous *GCK* mutation (83). Similar to control cells, these iPSCs differentiated into insulin-producing *β*-cells but showed reduced insulin secretion in response to glucose stimulation. In addition, iPSCs with two inactive *GCK* alleles also showed reduced differentiation efficiency recapitulating the functional impairment observed in patients and mouse models.

After the identification of *YIPF5* mutations causing a novel PNDM syndrome, Franco et al. characterized *in vitro* differentiated patient-derived iPSCs harboring a homozygous *YIPF5* mutation as well as genome-edited ESC in addition to a *β*-cell line (132). Functional impairment of YIPF5, responsible for trafficking between the endoplasmic reticulum and the Golgi apparatus, caused proinsulin retention at the ER resulting in ER stress-induced apoptosis and *β*-cell failure and, thus, diabetes.

In order to understand the role of activating mutations in *STAT3* during pancreatic development, iPSCs derived from patient fibroblasts were subjected to pancreatic differentiation and revealed a premature endocrine differentiation later preferentially forming *α*-cells which is in line with the observed phenotype of the patient (134). This defect results from enhanced nuclear localization of the mutant protein and NEUROG3 activation and could be rescued by correction of the *STAT3* mutation.

Inactivating mutations in *ABCC8* resulting in excess insulin secretion have been successfully employed for modeling congenital hyperinsulinism using ABCC8-deficient ESC (94, 95). In contrast, activating mutations in *ABCC8* have been described in diabetes (171). Thus, using the hPSC-systems, a better characterization of the components of the *β*-cell ATP-sensitive potassium channel may be obtained to understand the function of *β*-cells and associated pathomechanisms such as diabetes (caused by activating mutations) or the *vice-versa* effect of congenital hyperinsulinism (caused by inactivating mutations).

In the context of T2D susceptibility genes identified in GWAS, Zeng et al. generated ESC with null alleles for *KCNJ11*, also associated with MODY13 (96). Although the loss of KCNJ11 does not affect *in vitro* differentiation towards *β*-like cells and insulin production, these cells show impaired glucose-stimulated insulin secretion (96). In addition to hESCs with *KCNJ11* mutations, iPSCs have been generated from peripheral blood mononuclear cells with a heterozygous activating mutation in *KCNJ11* and are available for mechanistic studies as well as drug testing in differentiated pancreatic cells (97).

Skin fibroblasts from patients with Michell–Riley syndrome were used to generate iPSCs harboring a homozygous nonsense mutation in *RFX6* (140). Pancreatic differentiation revealed an impaired formation of pancreatic endoderm and thus, supports the impaired formation of endocrine cells in the pancreas in line with the patient phenotype.

In order to better understand the impact of different pathogenic *NEUROG3* variants, Zhang et al. expressed NEUROG3 mutant proteins at physiological levels in *NEUROG3* knockout ESC during pancreatic and intestinal differentiation determining the ability to rescue the generation of endocrine cells (139). Depending on the variant, expression resulted either in the decreased or abolished formation of pancreatic endocrine cells recapitulating the respective patient phenotype. Moreover, these effects could be retraced to be caused by impairment of NEUROG3 protein stability, DNA-binding affinity, and protein dimerization. Those features can differ in various tissues, a fact that emphasizes the importance of considering the relationship between protein structure and function.

Adenoviral *PAX4* overexpression during pancreatic differentiation of ESCs results in decreased glucagon-positive cells promoting the formation of monohormonal insulin-positive cells supporting its role in cell fate specification (92). This suggests a crucial role of intact *PAX4* in the development of healthy monohormonal insulin-positive cells.

A recent study reported the generation of iPSCs with a homozygous mutation in the *SLC2A2* gene (141). Peripheral mononuclear blood cells from a patient suffering from Fanconi–Bickel syndrome accompanied by early onset diabetes were reprogrammed using a non-integrating Sendai virus vector. These cells can be used to study the pathogenesis associated with defects in the GLUT2 glucose transporter in pancreatic *β*-cells.

In addition to KO mouse models, genome-engineered and patient-specific hPSCs have helped to get more insight into developmental and mechanistic processes as well as transcriptional networks (81). Not only do they provide additional information but sometimes even highlight the species-specific differences that make them even more crucial for a better understanding of monogenic diabetes.

A conditional *Hnf4a* KO in mice did not result in a diabetic phenotype but revealed that expression of the potassium channel subunit Kir6.2 regulating insulin secretion is promoted by *Hnf4a* (148). Patient-specific iPSCs inform about changes in transcriptional network (81). Similarly, *Hnf1a* mouse models with heterozygous KO present without developing diabetes, whereas a homozygous KO impairs *β*-cell function by reducing the insulin secretion. Stem cell-based models provided further details characterizing the developmental, transcriptional, and metabolic role of HNF1a (84). Furthermore, loss of *Wfs1* in mice showed impairment of *β*-cell development and function with a mild diabetic phenotype (149). Patient-specific iPSCs provided deeper insight into endoplasmatic reticulum stress and were used to test a possible therapeutic approach (142). Since GATA6 haploinsufficiency resulting in pancreatic agenesis in patients cannot be recapitulated in mice (172, 173), effects of GATA6 gene dosage on pancreatic differentiation *in vitro* helped in understanding the clinical presentation of different patients (135). Furthermore, hPSC models facilitate the characterization of disease-specific variants. For example, a *Hnf1b* heterozygous KO in mice is not associated with a diabetic phenotype (150), but patient-specific iPSC carrying a heterozygous variant of *HNF1B* helped to explain the MODY5 phenotype (90). In mice, *Neurog3* is essential for the development of the endocrine pancreas (144), but the disease phenotype slightly differs in humans (121). Expression of different disease-associated *NEUROG3* variants during *in vitro* differentiation helped in explaining various phenotypes in patients (139).

Taken together, a comparison of human and mouse model systems can provide further insight into the role of specific genes but also highlights the species-specific differences concerning, for example, transcription factor activity. That explains why human PSC-based models are crucially needed to compensate for those specific differences.

Therefore, genome editing in PSC or even patient-specific hiPSC provide a versatile approach to study developmental and functional effects of selected diabetes genes and variants and complement or even contradict data obtained from mouse models.

So far, many stem-cell-based models exist that characterize monogenic mutations resulting in early-onset diabetes. These models nicely elucidate the diabetic patient phenotype and help in understanding the common pathways in *β*-cell development and function. Altogether, improved screening for pathogenic variants in combination with thorough functional analysis will be the first step to precision medicine in diabetes therapy.

## Conclusion/Outlook

*In vitro* pancreatic differentiation of pluripotent stem cells is a powerful tool to better understand pancreatic development and the specific role of the involved transcription factors. Identification and characterization of specific variants in monogenic diabetes help in characterizing the complex transcriptional network and in overcoming phenotypic differences between patients and corresponding mouse models. In addition, these model systems provide the basis for drug development and testing that could benefit both patients of monogenic and multifactorial diabetes. Ideally, iPSCs of genetically disordered persons could be repaired and serve as a major source for tissue engineering and regeneration, *e.g. β*-cells in the case of monogenic diabetes.

Yet, some major roadblocks need to be kept in mind before translating genetically repaired iPSCs into clinics. First, epigenetic modifications of iPSCs which are derived from their originating tissue, might reduce their differentiation capacity and subsequent function as well as immune tolerance after autologous transplantation (174). Furthermore, genetic aberrations after reprogramming might bear tumorigenic potential and thus provoke carcinogenesis in the transplanted iPSC-derived tissues (174). Additionally, iPSC-derived tissues need to be manufactured according to SOPs and GMP guidelines which need lots of effort to implement those prerequisites into standard clinical care (175).

Altogether, these studies further urge the involvement of pluripotent stem cells in deciphering the underlying pathomechanisms as well as the affected genes, particularly when monogenic diabetes displays discrete clinical phenotypes and needs specific treatment depending on the subtype.

## Author Contributions

All authors wrote the paper. MM constructed figures. All authors contributed to the article and approved the submitted version.

## Funding

ANR-DFG collaborative research project (ANR-18-CE92-0031, DFG KL 2544/5-1) to CJ and AK; DFG funding with the identifiers KL 2544/6-1 (“Heisenberg-Programm”), KL 2544/7-1 (“Sachbeihilfe”) and KL 2544/1-2 (HEIST RTG) to AK; Agence Nationale pour la Recherche (ANR-09-GENO-021), the European Foundation for the Study of Diabetes/JDRF/Novo Nordisk, the Assistance Publique-Hôpitaux de Paris Programme Hospitalier de Recherche Clinique, and France Génomique (project DIAPED) to CJ. Novo Nordisk was not involved in the study design, collection, analysis, interpretation of data, the writing of this article or the decision to submit it for publication. MM received funding from Ulm University in the Clinician Scientist Program.

## Conflict of Interest

The authors declare that the research was conducted in the absence of any commercial or financial relationships that could be construed as a potential conflict of interest.

## Abbreviations

CRISPR, Clustered regularly interspaced short palindromic repeats; ER, Endoplasmatic reticulum; GWAS, Genome-wide association studies; hESC, Human embryonic stem cell; HLA, Human leucocyte antigen; hPSC, Human pluripotent stem cell; iPSC, Induced pluripotent stem cell; JOD, Juvenile-onset diabetes; KO, Knockout; MHC, Major histocompatibility complex; MODY, Maturity Onset of Diabetes in the Young; PNDM, Permanent neonatal diabetes mellitus; SNP, Single nucleotide polymorphism; T1D, Type 1 diabetes; T2D, Type 2 diabetes.
